# A Behavioral SIQR-B Model for Cholera Transmission: Impact of Adaptive Risk Perception on Disease Dynamics

**DOI:** 10.3390/epidemiologia7050142

**Published:** 2026-10-09

**Authors:** Pedro Pablo Cárdenas Alzate, Jaime Alberto Echeverri Franco, Jacobo Echeverri Hoyos

**Affiliations:** 1 Department of Mathematics and GEDNOL, Universidad Tecnológica de Pereira, Pereira 660003, Colombia; 2 Pulmonology, Clínica de Alta Tecnología, Oncólogos del Occidente, Pereira 660000, Colombia; jaime.echeverrif@gmail.com; 3 School of Medicine, Institución Universitaria Visión de las Américas, Pereira 660001, Colombia

**Keywords:** cholera, SIQR-B model, mathematical epidemiology, behavioral dynamics, risk perception, environmental transmission

## Abstract

**Background/Objectives**: Cholera remains a major public health problem, particularly in regions with limited access to safe drinking water and sanitation. Traditional compartmental models may underrepresent adaptive human behavioral responses during outbreaks. This study aims to investigate the epidemiological effects of adaptive risk perception within a deterministic SIQR-B framework for cholera transmission. **Methods**: We developed a deterministic SIQR-B model incorporating adaptive behavior through a nonlinear risk-perception function that modulates environmental transmission. The model includes susceptible, infected, quarantined, and recovered individuals together with the environmental concentration of *Vibrio cholerae*. Positivity, boundedness, equilibria, the basic reproduction number, and stability were analyzed, complemented by numerical simulations and robustness analysis across alternative transmission regimes. **Results**: Under the baseline parameter setting, adaptive behavior reduced the infected peak by 19.03% and delayed its occurrence by approximately 1.13 days. In contrast, cumulative incidence over the 200-day simulation horizon decreased by only approximately 0.033%. Additional simulations across alternative transmission regimes preserved the qualitative effect of adaptive behavior on peak mitigation and epidemic timing, although the magnitude of these effects varied with the transmission configuration and behavioral intensity. **Conclusions**: Within the proposed theoretical framework, adaptive behavioral feedback primarily mitigates short-term peak burden and alters epidemic timing rather than substantially reducing cumulative transmission. These findings highlight the importance of incorporating behavioral responses into mathematical models of cholera dynamics while recognizing that the quantitative effects depend on the assumed parameter regime.

## 1. Introduction

Cholera is a highly virulent waterborne disease caused by the bacterium *Vibrio cholerae*, and it continues to pose a serious threat to public health in developing regions with limited access to safe drinking water and sanitation. Despite significant advances in medical treatment and prevention, recurring outbreaks in countries such as Haiti and Syria highlight the persistent vulnerability of affected populations. Recent epidemiological data confirm that cholera remains a major global public-health challenge. According to the World Health Organization (WHO), more than 560,000 cholera cases and over 6000 associated deaths were reported across 60 countries in 2024, with a substantial increase in mortality compared with 2023 [1]. These figures highlight the persistent global burden of cholera and reinforce the need for mathematical frameworks capable of examining environmental transmission and adaptive population responses.

Mathematical modeling has played an important role in understanding the transmission dynamics of infectious diseases such as cholera. Classic compartmental models, such as the SIR, SEIR, and their variants, have been widely used to describe the evolution of epidemics [2,3]. With regard to cholera, models that incorporate environmental reservoirs, commonly known as SIQR-B or SIR-B models, have proven particularly relevant, as they account for the concentration of bacteria in contaminated water sources [4,5].

The present formulation retains the basic compartmental structure shared with classical SIR-type models, namely the progression of individuals through susceptible, infected, and recovered states. However, it extends this structure by explicitly incorporating quarantine and an environmental bacterial reservoir, both of which are particularly relevant to cholera dynamics. The behavioral component introduced here further modifies the environmental force of infection through a saturating risk-perception mechanism. Thus, the proposed model should be interpreted as an extension of established compartmental cholera frameworks rather than as an entirely new compartmental paradigm.

Although adaptive human behavior has been incorporated into several cholera transmission models, important mathematical and epidemiological questions remain open [6,7]. Previous studies have shown that behavioral responses may substantially modify cholera dynamics through changes in contact rates, awareness mechanisms, and adaptive responses to disease risk [6]. These contributions demonstrate that behavioral epidemiology has become an active area of research rather than a neglected aspect of cholera modeling.

Motivated by these previous studies, we propose a behavioral SIQR-B model in which adaptive risk perception modifies the environmental force of infection through a nonlinear incidence function. The novelty of the present work does not lie in introducing behavioral responses themselves, but in analyzing their epidemiological consequences within an SIQR-B framework that explicitly tracks an isolation/case-management compartment together with an environmental bacterial reservoir. In the present formulation, the *Q* compartment does not generate independent feedback on the transmission subsystem (S,I,B); rather, it records the burden of infected individuals removed from active transmission through isolation. Accordingly, the transmission-level effect of the quarantine rate γ operates through the total removal rate from the infectious compartment. Specifically, we derive the basic reproduction number, establish positivity and boundedness of solutions; investigate the local and global stability of the disease-free equilibrium; analyze the existence of the endemic equilibrium; and illustrate the qualitative effects of adaptive behavior through numerical simulations. Accordingly, the present work should be interpreted as a qualitative theoretical modeling study rather than as an outbreak-specific predictive or data-calibrated epidemiological model.

## 2. Analysis of the Mathematical Model

To describe the transmission dynamics of cholera, we propose a deterministic compartmental model that extends the classical SIQR-B framework by incorporating adaptive human behavior.

The total human population at time *t*, denoted by N(t), is divided into four compartments:

- S(t): susceptible individuals;
- I(t): infected individuals;
- Q(t): quarantined/isolated infected individuals under effective case-management conditions.
- R(t): recovered individuals.

In the present formulation, the term “quarantine” is used in the mathematical sense to denote infected individuals who are effectively isolated from the general population. It should therefore be interpreted as an isolation/case-management compartment rather than as quarantine of susceptible contacts.

Additionally, the model includes an environmental compartment:

- B(t): concentration of *Vibrio cholerae* in contaminated water.

Thus, the total population is given by

(1) N(t)=S(t)+I(t)+Q(t)+R(t).

### 2.1. Behavioral Assumption

Unlike classical models, we assume that the transmission rate is influenced by adaptive human behavior. As the number of infected individuals increases, awareness and preventive actions, such as improved hygiene and reduced exposure, also increase, leading to a reduction in the effective transmission rate.

To model this effect, we introduce a behavior-dependent function ω(I) satisfying

(2) $$0\leq \omega \left(I\right)<1,\;\omega^{\prime }\left(I\right)>0.$$

A typical functional form is given by

(3) $$\omega \left(I\right)=\frac{aI}{1+bI},$$

where a,b>0 are parameters representing the intensity and saturation of the behavioral response, respectively. We assume a≤b, which ensures that 0≤ω(I)<1 for all I≥0.

### 2.2. Force of Infection

The transmission of cholera occurs through contact with contaminated water. Therefore, the force of infection is defined as

(4) $$\lambda \left(I,B\right)=\left(1-\omega \left(I\right)\right)\;\beta_{e}\frac{B}{k+B},$$

where $\beta_{e}$ is the exposure rate to the bacteria and *k* is the half-saturation constant.

### 2.3. Model Equations

The dynamics of the system are governed by the following nonlinear system of differential equations:

(5) $$\left\{\begin{array}{cc} \frac{dS}{dt} & =\Lambda -\lambda (I,B)S-\mu S,\\ \frac{dI}{dt} & =\lambda (I,B)S-(\alpha +\gamma +\delta +\mu )I,\\ \frac{dQ}{dt} & =\gamma I-(\eta +\delta +\mu )Q,\\ \frac{dR}{dt} & =\alpha I+\eta Q-\mu R,\\ \frac{dB}{dt} & =\xi I-\sigma B. \end{array}\right.$$

To ensure clarity and facilitate interpretation, Table 1 presents the model parameters together with their biological and epidemiological meanings.

The environmental variable *B* is expressed as bacterial concentration; therefore, *k* has the same units as *B*, while ξ represents bacterial concentration produced per infected individual per unit time. The parameter values in Table 1 define an illustrative baseline scenario and should not be interpreted as a calibration to a specific cholera outbreak. The demographic, epidemiological, and environmental parameters were selected to provide a mathematically consistent qualitative transmission regime, with their interpretation guided by established cholera models incorporating environmental reservoirs and saturating waterborne transmission [5,6]. Because the scaling of the environmental bacterial variable differs among model formulations, the numerical values of $\beta_{e}$, *k*, ξ, and σ are model-dependent and are not intended as directly transferable empirical estimates. The behavioral parameters *a* and *b* are likewise illustrative values satisfying the admissibility condition a≤b and are further examined in the robustness analysis. Consequently, the numerical results are intended for qualitative comparison and robustness assessment rather than outbreak-specific prediction.

### 2.4. Model Description

The state variables of the model are S(t), I(t), Q(t), R(t), and B(t), representing susceptible individuals, infected individuals, quarantined/isolated infected individuals, recovered individuals, and the environmental concentration of *Vibrio cholerae*, respectively.

The model incorporates the following mechanisms:

- Susceptible individuals become infected through environmental exposure;
- Infected individuals may recover, die, or enter quarantine;
- Quarantined individuals do not contribute to environmental contamination;
- The bacterial concentration increases due to infected individuals and decreases naturally over time;
- Behavioral adaptation reduces the effective exposure to contaminated water.

The present formulation assumes that quarantined individuals are effectively isolated and therefore do not contribute to the environmental bacterial reservoir. In addition, adaptive behavioral responses are assumed to reduce exposure to contaminated water without directly modifying bacterial shedding or recovery rates. These assumptions are consistent with the intended qualitative analysis of the proposed model and provide a mathematically tractable framework for investigating the epidemiological impact of behavioral adaptation. The model assumes homogeneous mixing of the human population, constant recruitment and natural mortality rates, and environmental transmission as the only transmission route. Recovered individuals are assumed to remain immune over the epidemic time horizon considered. These assumptions are adopted to maintain model tractability and should be regarded as limitations when interpreting the results.

### 2.5. Remark

The inclusion of the function ω(I) provides a specific nonlinear feedback mechanism linking epidemic severity with adaptive human behavioral response within the proposed SIQR-B framework.

### 2.6. Positivity and Boundedness of Solutions

In epidemiological models, it is essential to guarantee that all state variables remain nonnegative for all future times. In this section, we prove that the solutions of system (5) are positive and bounded in a biologically feasible region.

The positivity argument follows standard invariant-region techniques for compartmental epidemiological models [8].

**Theorem** **1.** *Let $\left(S\left(0\right),I\left(0\right),Q\left(0\right),R\left(0\right),B\left(0\right)\right)\in R_{+}^{5}$. Then every solution of system *(5)* remains nonnegative for all t≥0.*

**Proof.**  From the first equation of system (5), we have

$$\frac{dS}{dt}=\Lambda -\lambda \left(I,B\right)S-\mu S.$$

Since λ(I,B)≥0, it follows that

$$\frac{dS}{dt}\geq -\left(\lambda \left(I,B\right)+\mu \right)S.$$

Therefore,

$$S\left(t\right)\geq S\left(0\right)\exp\left(-\int_{0}^{t}\left(\lambda \left(I\left(s\right),B\left(s\right)\right)+\mu \right)\;ds\right)\geq 0.$$

Similarly, for the infected compartment,

$$\frac{dI}{dt}=\lambda \left(I,B\right)S-\left(\alpha +\gamma +\delta +\mu \right)I,$$

which implies

$$\frac{dI}{dt}\geq -\left(\alpha +\gamma +\delta +\mu \right)I.$$

Hence,

$$I\left(t\right)\geq I\left(0\right)e^{-(\alpha +\gamma +\delta +\mu )t}\geq 0.$$

For the quarantined compartment,

$$\frac{dQ}{dt}=\gamma I-\left(\eta +\delta +\mu \right)Q\geq -\left(\eta +\delta +\mu \right)Q,$$

and therefore

$$Q\left(t\right)\geq Q\left(0\right)e^{-(\eta +\delta +\mu )t}\geq 0.$$

For the recovered compartment,

$$\frac{dR}{dt}=\alpha I+\eta Q-\mu R\geq -\mu R,$$

so that

$$R\left(t\right)\geq R\left(0\right)e^{-\mu t}\geq 0.$$

Finally, for the environmental bacterial concentration,

$$\frac{dB}{dt}=\xi I-\sigma B\geq -\sigma B.$$

Thus,

$$B\left(t\right)\geq B\left(0\right)e^{-\sigma t}\geq 0.$$

Consequently, all state variables remain nonnegative for all t≥0.    □

The boundedness argument is based on standard differential-inequality and comparison techniques commonly used in epidemiological models [8].

**Theorem** **2.** *All nonnegative solutions of system *(5)* are bounded. Moreover, the region*

$$\Omega =\left\{\left(S,I,Q,R,B\right)\in R_{+}^{5}:S+I+Q+R\leq \frac{\Lambda }{\mu },\;B\leq \frac{\xi \Lambda }{\mu \sigma }\right\}$$

*is positively invariant.*

**Proof.**  Let

N(t)=S(t)+I(t)+Q(t)+R(t).

Adding the equations for the human compartments gives

$$\frac{dN}{dt}=\Lambda -\mu N-\delta \left(I+Q\right)\leq \Lambda -\mu N.$$

Hence, by the comparison principle,

$$N\left(t\right)\leq N\left(0\right)e^{-\mu t}+\frac{\Lambda }{\mu }\left(1-e^{-\mu t}\right).$$

Defining

$$M=\max\left\{N\left(0\right),\frac{\Lambda }{\mu }\right\},$$

we obtain

0≤N(t)≤M,t≥0,

and therefore

0≤I(t)≤M.

From the environmental equation,

$$\frac{dB}{dt}=\xi I-\sigma B\leq \xi M-\sigma B.$$

A second application of the comparison principle yields

$$0\leq B\left(t\right)\leq B\left(0\right)e^{-\sigma t}+\frac{\xi M}{\sigma }\left(1-e^{-\sigma t}\right)\leq \max\left\{B\left(0\right),\frac{\xi M}{\sigma }\right\}.$$

Thus, every nonnegative solution of system (5) is bounded.

Furthermore,

$$\underset{t\to \infty }{\lim\;\sup}\;N\left(t\right)\leq \frac{\Lambda }{\mu }.$$

Consequently, for every ε>0, there exists $T_{\varepsilon }>0$ such that

$$I\left(t\right)\leq N\left(t\right)\leq \frac{\Lambda }{\mu }+\varepsilon ,\;t\geq T_{\varepsilon }.$$

For $t\geq T_{\varepsilon }$,

$$\frac{dB}{dt}\leq \xi \left(\frac{\Lambda }{\mu }+\varepsilon \right)-\sigma B,$$

and therefore

$$\underset{t\to \infty }{\lim\;\sup}\;B\left(t\right)\leq \frac{\xi }{\sigma }\left(\frac{\Lambda }{\mu }+\varepsilon \right).$$

Since ε>0 is arbitrary,

$$\underset{t\to \infty }{\lim\;\sup}\;B\left(t\right)\leq \frac{\xi \Lambda }{\mu \sigma }.$$

Finally, if the initial condition belongs to Ω, then on the boundary N=Λ/μ,

$$\frac{dN}{dt}=-\delta \left(I+Q\right)\leq 0,$$

while on the boundary B=ξΛ/(μσ),

$$\frac{dB}{dt}=\xi I-\frac{\xi \Lambda }{\mu }\leq 0,$$

because I≤N≤Λ/μ. Together with the nonnegativity of the solutions, these inequalities show that Ω is positively invariant.    □

### 2.7. Disease-Free Equilibrium and Basic Reproduction Number

In this section, we determine the disease-free equilibrium (DFE) of the model and compute the basic reproduction number using the next-generation matrix approach [9].

**Theorem** **3.** *The disease-free equilibrium of system *(5) *is given by*

$$E_{0}=\left(\frac{\Lambda }{\mu },0,0,0,0\right).$$

**Proof.**  The disease-free equilibrium is obtained by setting I=Q=R=B=0 in system (5) and solving the remaining equations at equilibrium. From dS/dt=0, we obtain Λ−μS=0. Hence, S=Λ/μ. Therefore, the disease-free equilibrium is

$$E_{0}=\left(\frac{\Lambda }{\mu },0,0,0,0\right).$$

   □

To compute the basic reproduction number $R_{0}$, we employ the next-generation matrix approach developed by van den Driessche and Watmough [9] and based on the general framework introduced by Diekmann et al. [10]. According to this method, only those compartments that actively participate in the generation or maintenance of infection are included in the infected subsystem. In the present model, these compartments are (I,B), where *I* represents infectious individuals and *B* denotes the environmental concentration of *Vibrio cholerae*, which mediates indirect transmission. The corresponding vectors of new infection terms and transition terms are therefore defined as follows:

$$F=\left(\begin{array}{c} \lambda (I,B)S\\ 0 \end{array}\right),$$

and the transition terms are

$$V=\left(\begin{array}{c} (\alpha +\gamma +\delta +\mu )I\\ \sigma B-\xi I \end{array}\right).$$

Evaluating at the disease-free equilibrium and noting that ω(0)=0, the Jacobian matrices become

$$F=\left(\begin{array}{cc} 0 & \frac{\beta_{e}\Lambda }{k\mu }\\ 0 & 0 \end{array}\right),\;V=\left(\begin{array}{cc} \alpha +\gamma +\delta +\mu & 0\\ -\xi & \sigma \end{array}\right).$$

The next-generation matrix is defined by $K=FV^{-1}$. After straightforward calculations, the basic reproduction number is obtained as

$$R_{0}=\frac{\beta_{e}\xi \Lambda }{k\mu \sigma (\alpha +\gamma +\delta +\mu )}.$$

### 2.8. Local Stability of the Disease-Free Equilibrium

In this section, we analyze the local stability of the disease-free equilibrium $E_{0}$.

The Jacobian matrix associated with system (5) is given by

$$J=\left(\begin{array}{ccccc} -\lambda (I,B)-\mu & -\frac{\partial \lambda }{\partial I}S & 0 & 0 & -\frac{\partial \lambda }{\partial B}S\\ \lambda (I,B) & \frac{\partial \lambda }{\partial I}S-\left(\alpha +\gamma +\delta +\mu \right) & 0 & 0 & \frac{\partial \lambda }{\partial B}S\\ 0 & \gamma & -(\eta +\delta +\mu ) & 0 & 0\\ 0 & \alpha & \eta & -\mu & 0\\ 0 & \xi & 0 & 0 & -\sigma \end{array}\right).$$

Evaluating the Jacobian matrix at the disease-free equilibrium and using the fact that ω(0)=0, we obtain

$$J\left(E_{0}\right)=\left(\begin{array}{ccccc} -\mu & 0 & 0 & 0 & -\frac{\beta_{e}\Lambda }{k\mu }\\ 0 & -(\alpha +\gamma +\delta +\mu ) & 0 & 0 & \frac{\beta_{e}\Lambda }{k\mu }\\ 0 & \gamma & -(\eta +\delta +\mu ) & 0 & 0\\ 0 & \alpha & \eta & -\mu & 0\\ 0 & \xi & 0 & 0 & -\sigma \end{array}\right).$$

**Theorem** **4.** *The disease-free equilibrium $E_{0}$ is locally asymptotically stable if $R_{0}<1$, and unstable if $R_{0}>1$.*

**Proof.**  The local dynamics around the disease-free equilibrium are determined by the eigenvalues of the Jacobian matrix $J(E_{0})$. Since the basic reproduction number $R_{0}$ represents the spectral radius of the next-generation matrix, it follows from the theory of van den Driessche and Watmough that all eigenvalues have negative real parts when $R_{0}<1$. Therefore, the disease-free equilibrium is locally asymptotically stable. On the other hand, if $R_{0}>1$, then at least one eigenvalue has a positive real part, implying instability of the disease-free equilibrium.    □

Nonlinear incidence functions are frequently used in epidemiological models to capture saturation and behavioral effects [11].

### 2.9. Endemic Equilibrium

In this section, we determine the endemic equilibrium of system (5), corresponding to the persistent presence of cholera in the population. Let the endemic equilibrium be denoted by $E^{*}=\left(S^{*},I^{*},Q^{*},R^{*},B^{*}\right)$, where all components are positive. At equilibrium, we impose

$$\frac{dS}{dt}=\frac{dI}{dt}=\frac{dQ}{dt}=\frac{dR}{dt}=\frac{dB}{dt}=0.$$

The proposed model is formulated as an extension of the classical SIQR-B framework for cholera transmission. The compartments *S*, *I*, *Q*, and *R* retain their standard epidemiological interpretation, while *B* represents the environmental bacterial reservoir. The main modification considered here is the incorporation of the adaptive behavioral response ω(I) into the environmental force of infection. Thus, rather than introducing a new compartmental structure, the model reformulates the transmission mechanism so that environmental exposure decreases as the perceived epidemic risk increases. This allows the interaction between quarantine, environmental transmission, and adaptive behavior to be studied within a unified framework. Thus, system (5) becomes

$$0=\Lambda -\lambda \left(I^{*},B^{*}\right)S^{*}-\mu S^{*},$$

$$0=\lambda \left(I^{*},B^{*}\right)S^{*}-\left(\alpha +\gamma +\delta +\mu \right)I^{*},$$

$$0=\gamma I^{*}-\left(\eta +\delta +\mu \right)Q^{*},$$

$$0=\alpha I^{*}+\eta Q^{*}-\mu R^{*},$$

$$0=\xi I^{*}-\sigma B^{*}.$$

From the third equation, we obtain

$$Q^{*}=\frac{\gamma I^{*}}{\eta +\delta +\mu }.$$

From the fifth equation,

$$B^{*}=\frac{\xi I^{*}}{\sigma }.$$

Substituting these expressions into the remaining equations yields the reduced endemic equilibrium system. Moreover, from the second equilibrium equation, we obtain

$$\lambda \left(I^{*},B^{*}\right)S^{*}=\left(\alpha +\gamma +\delta +\mu \right)I^{*}.$$

Hence,

$$S^{*}=\frac{\left(\alpha +\gamma +\delta +\mu \right)I^{*}}{\lambda (I^{*},B^{*})}.$$

Using the expression of the force of infection together with the behavioral response function, the endemic equilibrium satisfies a nonlinear algebraic system whose existence depends on the condition $R_{0}>1$.

**Theorem** **5.** *If $R_{0}>1$, then system *(5) *admits at least one endemic equilibrium. Moreover, if a≤b, this endemic equilibrium is unique.*

**Proof.**  Let

D=α+γ+δ+μ.

At an endemic equilibrium, $I^{*}>0$, and from the equilibrium equations,

$$B^{*}=\frac{\xi I^{*}}{\sigma },\;Q^{*}=\frac{\gamma I^{*}}{\eta +\delta +\mu }.$$

Adding the first two equilibrium equations gives

$$S^{*}=\frac{\Lambda -DI^{*}}{\mu },$$

and therefore

$$0<I^{*}<\frac{\Lambda }{D}.$$

Since

$$1-\omega \left(I^{*}\right)=1-\frac{aI^{*}}{1+bI^{*}}=\frac{1+\left(b-a\right)I^{*}}{1+bI^{*}},$$

substitution into the infected equilibrium equation yields

(6) $$\beta_{e}\xi \left[1+\left(b-a\right)I^{*}\right]\left(\Lambda -DI^{*}\right)=D\mu \left(\sigma k+\xi I^{*}\right)\left(1+bI^{*}\right).$$

Define

$$G\left(I\right)=\beta_{e}\xi \left[1+\left(b-a\right)I\right]\left(\Lambda -DI\right)-D\mu \left(\sigma k+\xi I\right)\left(1+bI\right).$$

At I=0,

$$G\left(0\right)=D\mu \sigma k\left(R_{0}-1\right)>0$$

whenever $R_{0}>1$, whereas

$$G\left(\frac{\Lambda }{D}\right)=-D\mu \left(\sigma k+\frac{\xi \Lambda }{D}\right)\left(1+\frac{b\Lambda }{D}\right)<0.$$

Hence, by continuity, there exists at least one

$$I^{*}\in \left(0,\frac{\Lambda }{D}\right)$$

such that $G(I^{*})=0$. Therefore, at least one biologically meaningful endemic equilibrium exists whenever $R_{0}>1$.

Furthermore, if a≤b, then

$$G^{\prime \prime }\left(I\right)=-2\beta_{e}\xi D\left(b-a\right)-2D\mu b\xi <0.$$

Thus, *G* is strictly concave on the biologically feasible interval. Since G(0)>0 and G(Λ/D)<0, it can cross zero only once in (0,Λ/D). Therefore, the endemic equilibrium is unique when a≤b.    □

**Remark** **1.** *The preceding theorem establishes the existence of an endemic equilibrium for $R_{0}>1$ and its uniqueness under the stated condition. A complete local or global stability analysis of the endemic equilibrium is beyond the scope of the present study and remains an interesting direction for future work.*

### 2.10. Global Stability of the Disease-Free Equilibrium

In this section, we investigate the global asymptotic stability of the disease-free equilibrium $E_{0}$.

**Theorem** **6.** *If $R_{0}<1$, then the disease-free equilibrium $E_{0}=\left(\Lambda /\mu ,0,0,0,0\right)$ is globally asymptotically stable in the feasible region* Ω*.*

**Proof.**  Consider the Lyapunov function

$$V\left(I,B\right)=I+\frac{\beta_{e}\Lambda }{k\mu \sigma }B.$$

The choice of a Lyapunov function involving only *I* and *B* is motivated by the structure of the transmission subsystem. These two variables represent the epidemiologically active components responsible for generating new infections. Once *I* and *B* vanish, the remaining variables form a downstream subsystem: *Q* and *R* decay asymptotically to zero, while *S* converges to Λ/μ. Therefore, controlling the (I,B) subsystem is sufficient to establish convergence of the full system to the disease-free equilibrium through LaSalle’s invariance principle [12].

Clearly, V(I,B)≥0 for all (I,B)∈Ω, and V(I,B)=0 if and only if I=B=0. Differentiating *V* along the solutions of system (5), we obtain

$$\frac{dV}{dt}=\frac{dI}{dt}+\frac{\beta_{e}\Lambda }{k\mu \sigma }\frac{dB}{dt}.$$

Substituting the model equations yields

$$\frac{dV}{dt}=\lambda \left(I,B\right)S-\left(\alpha +\gamma +\delta +\mu \right)I+\frac{\beta_{e}\Lambda }{k\mu \sigma }\left(\xi I-\sigma B\right).$$

Using the fact that S≤Λ/μ, together with

$$\lambda \left(I,B\right)=\left(1-\omega \left(I\right)\right)\frac{\beta_{e}B}{k+B},$$

and observing that

$$\frac{B}{k+B}\leq \frac{B}{k},$$

we obtain

$$\frac{dV}{dt}\leq \frac{\beta_{e}\Lambda }{k\mu }B-\left(\alpha +\gamma +\delta +\mu \right)I+\frac{\beta_{e}\Lambda \xi }{k\mu \sigma }I-\frac{\beta_{e}\Lambda }{k\mu }B.$$

Hence,

$$\frac{dV}{dt}\leq \left(\frac{\beta_{e}\Lambda \xi }{k\mu \sigma }-\left(\alpha +\gamma +\delta +\mu \right)\right)I.$$

Using the definition of $R_{0}$, we conclude that

$$\frac{dV}{dt}\leq \left(\alpha +\gamma +\delta +\mu \right)\left(R_{0}-1\right)I.$$

Therefore, if $R_{0}<1$, then

$$\frac{dV}{dt}\leq 0.$$

Moreover,

$$\frac{dV}{dt}=0$$

holds only if

I=0andB=0.

When $\dot{V}=0$, we obtain I=B=0. Consequently,

$$\frac{dQ}{dt}=-\left(\eta +\delta +\mu \right)Q,$$

so that Q(t)→0. Moreover,

$$\frac{dR}{dt}=\eta Q-\mu R,$$

which implies R(t)→0. Finally,

$$\frac{dS}{dt}=\Lambda -\mu S,$$

and therefore

$$S\left(t\right)\to \frac{\Lambda }{\mu }.$$

Thus, every trajectory contained in the set where $\dot{V}=0$ approaches the disease-free equilibrium

$$E_{0}=\left(\frac{\Lambda }{\mu },0,0,0,0\right).$$

Hence, by LaSalle’s invariance principle, $E_{0}$ is globally asymptotically stable whenever $R_{0}<1$.

This result does not contradict Remark 1. Remark 1 concerns the unresolved stability of the endemic equilibrium when $R_{0}>1$, whereas the present Lyapunov–LaSalle argument establishes global stability of the disease-free equilibrium under the distinct threshold condition $R_{0}<1$.    □

Lyapunov methods are classical tools for studying global stability in epidemiological systems [8].

### 2.11. Sensitivity Analysis of the Basic Reproduction Number

To better understand the epidemiological importance of the model parameters, we analyze the sensitivity of the basic reproduction number $R_{0}$. The normalized forward sensitivity index of $R_{0}$ with respect to a parameter *p* is defined as

$$\Upsilon_{p}^{R_{0}}=\frac{\partial R_{0}}{\partial p}\cdot \frac{p}{R_{0}}.$$

Recall that

(7) $$R_{0}=\frac{\beta_{e}\xi \Lambda }{k\mu \sigma (\alpha +\gamma +\delta +\mu )}.$$

The next-generation decomposition adopted here treats bacterial shedding into the environmental compartment as a transition within the infected subsystem rather than as a new host-infection event. This convention leads to the product-form reproduction number given above. Alternatively, if environmental bacterial production is classified as a new infection event, the next-generation approach yields the square-root form of the corresponding quantity. Both conventions are epidemiologically equivalent with respect to the invasion threshold, since they lead to the same threshold condition $R_{0}=1$.

For the baseline parameter values listed in Table 1, the product-form threshold is

$$R_{0}\approx 3.33\times 10^{5},$$

while the corresponding square-root convention gives approximately 577.19. These large values indicate that the baseline parameter set represents a high-transmission illustrative regime and should not be interpreted as an empirical estimate of the reproduction number for a specific cholera outbreak. This observation further motivates the robustness analysis across alternative transmission regimes presented below.

The sensitivity index measures the relative change in $R_{0}$ produced by a relative change in the parameter *p*. For the environmental transmission rate $\beta_{e}$, we obtain

$$\Upsilon_{\beta_{e}}^{R_{0}}=\frac{\partial R_{0}}{\partial \beta_{e}}\cdot \frac{\beta_{e}}{R_{0}}=1.$$

Thus, a 10% increase in $\beta_{e}$ produces a 10% increase in $R_{0}$. Similarly, for the bacterial shedding rate ξ,

$$\Upsilon_{\xi }^{R_{0}}=1.$$

On the other hand, for the bacterial decay rate σ,

$$\Upsilon_{\sigma }^{R_{0}}=-1,$$

which indicates that increasing bacterial decay reduces the transmission potential of cholera. For the quarantine rate γ,

$$\Upsilon_{\gamma }^{R_{0}}=-\frac{\gamma }{\alpha +\gamma +\delta +\mu }.$$

The remaining normalized sensitivity indices of $R_{0}$ are

$$\Upsilon_{\Lambda }^{R_{0}}=1,\;\Upsilon_{k}^{R_{0}}=-1,$$

$$\Upsilon_{\alpha }^{R_{0}}=-\frac{\alpha }{\alpha +\gamma +\delta +\mu },\;\Upsilon_{\delta }^{R_{0}}=-\frac{\delta }{\alpha +\gamma +\delta +\mu },$$

and

$$\Upsilon_{\mu }^{R_{0}}=-1-\frac{\mu }{\alpha +\gamma +\delta +\mu }.$$

Thus, $R_{0}$ is directly proportional to Λ and inversely proportional to *k*, while increases in α, δ, and μ reduce the reproduction number. The behavioral parameters *a* and *b* do not appear explicitly in $R_{0}$ because ω(0)=0; consequently, their influence is expressed through the nonlinear epidemic dynamics rather than through the invasion threshold itself.

Within the present model, increasing the isolation rate γ reduces $R_{0}$ through its contribution to the total removal rate α+γ+δ+μ. The sensitivity analysis also shows that environmental transmission and bacterial persistence parameters directly influence the invasion threshold. In particular, increasing the bacterial decay rate σ decreases $R_{0}$, whereas the effect of γ should be interpreted as part of the combined removal process rather than as an independent transmission mechanism.

It is important to emphasize that the behavioral parameters *a* and *b* do not appear explicitly in $R_{0}$. This occurs because the reproduction number is evaluated at the disease-free equilibrium, where I=0 and therefore ω(0)=0. Consequently, adaptive behavior does not modify the initial invasion threshold of the model. Instead, behavioral feedback becomes active once infections are present and influences the subsequent transient epidemic dynamics, including the epidemic peak and its timing.

## 3. Numerical Simulations

In this section, we present numerical simulations to illustrate the dynamical behavior of the proposed SIQR-B cholera model with adaptive human behavior. The parameter values employed in these simulations were selected from published studies whenever epidemiological estimates were available. For parameters lacking direct experimental or epidemiological estimates, biologically plausible values were adopted to explore the qualitative behavior of the model. Consequently, the numerical results should be interpreted as illustrative analyses of the influence of adaptive behavioral responses rather than as a calibrated reconstruction of a specific cholera outbreak. The main objective is to analyze how behavioral response affects the spread of cholera and the environmental concentration of *Vibrio cholerae*. The baseline parameter values are summarized in Table 1. These baseline values are used solely to generate a qualitative transmission regime for comparative simulations. They are not intended to reproduce an empirically estimated reproduction number or the demographic equilibrium of a particular population. Accordingly, absolute numerical magnitudes should not be interpreted as outbreak-specific epidemiological estimates. For reproducibility, the revised numerical analysis explicitly reports the initial conditions, simulation interval, and numerical integration method used in each simulation.

It should be noted that the disease-free demographic equilibrium satisfies $S_{0}=\Lambda /\mu =100,000$, whereas the numerical simulations are initialized at S(0)=10,000. This difference is intentional: the simulations start away from the demographic equilibrium and are designed to illustrate transient epidemic dynamics rather than to represent a population initially at the disease-free demographic steady state.

For reference, if the susceptible population is held fixed at a generic level *S*, the corresponding low-infection, low-environmental-bacteria transmission quantity is

$$T\left(S\right)=\frac{\beta_{e}\xi S}{k\sigma (\alpha +\gamma +\delta +\mu )}.$$

Hence, because S(0)=0.1(Λ/μ) in the baseline simulation, $T\left(S\left(0\right)\right)=0.1R_{0}$. This quantity is introduced only to characterize the transmission potential associated with the initial susceptible level; it is not interpreted as an exact effective reproduction number at t=0, because I(0)>0, B(0)>0, and the behavioral response is already active.

S(0)=10,000,I(0)=10,Q(0)=0,R(0)=0,B(0)=50.

To distinguish changes in epidemic peak burden from changes in total transmission over the simulation horizon, we additionally define the cumulative incidence C(t) through

(8) $$\frac{dC}{dt}=\lambda \left(I,B\right)S,\;C\left(0\right)=0.$$

The variable C(t) is used only as an auxiliary outcome measure and does not modify the dynamics of system (5).

The numerical system was integrated over the interval t∈[0,200] days using the explicit Runge–Kutta method of order 5(4) (RK45) implemented in the Python 3.13.5 function solve_ivp. The relative and absolute error tolerances were set to $10^{-8}$ and $10^{-10}$, respectively. For each simulation, the infected peak was defined as the maximum value of I(t) over the 200-day simulation interval, and the corresponding peak time was identified as the time at which this maximum was attained.

The baseline behavioral parameters were a=0.005 and b=0.01. The behavioral response function is given by

(9) $$\omega \left(I\right)=\frac{aI}{1+bI},$$

where *a* represents the intensity of the behavioral response and *b* represents the saturation level of such response.

To evaluate the impact of adaptive behavior, we compare two scenarios:

- **Scenario 1: No behavioral response**, corresponding to a=0, and hence ω(I)=0;
- **Scenario 2: Adaptive behavioral response**, corresponding to a>0, where the effective transmission rate decreases as the number of infected individuals increases.

In the absence of behavioral response, susceptible individuals remain highly exposed to contaminated environments, leading to a faster epidemic growth and a higher infection peak. In contrast, when adaptive behavior is incorporated, the effective force of infection decreases as awareness increases, producing a reduction in the epidemic peak and a delay in the outbreak progression. Therefore, the numerical simulations support the theoretical results and show that behavioral adaptation may play a significant role in mitigating cholera transmission.

**Figure 1:**
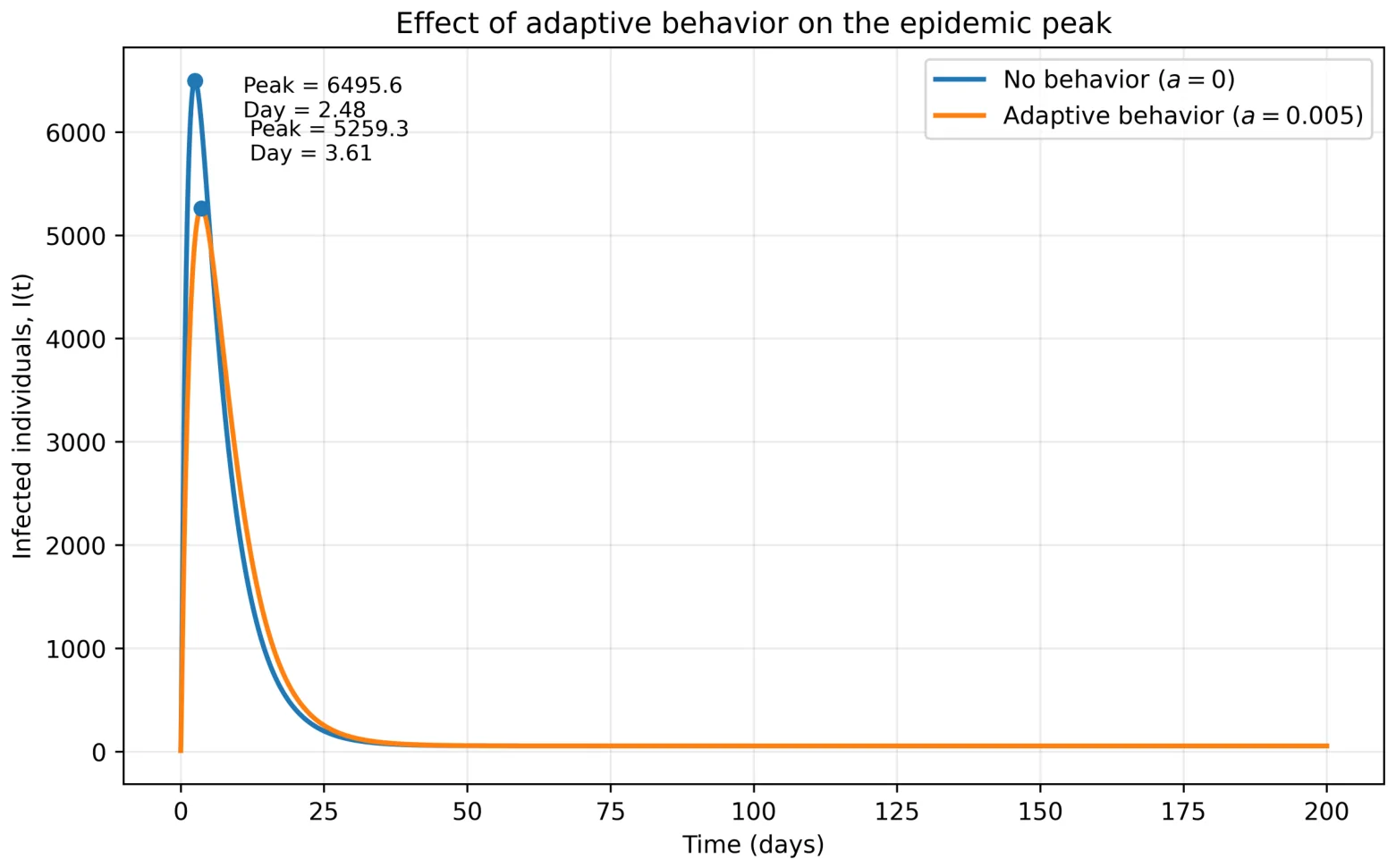
Direct comparison of the infected population I(t) under no behavioral response (a=0) and adaptive behavioral response (a=0.005), with b=0.01 individual^−1^. All remaining parameters are fixed at the baseline values in Table 1, with initial conditions S(0)=10,000, I(0)=10, Q(0)=R(0)=0, and B(0)=50. Adaptive behavior reduces the infection peak from 6495.61 to 5259.30 individuals and delays the peak from day 2.48 to day 3.61.

Figure 1 provides a direct comparison of the infected population under the no-behavior and adaptive-behavior scenarios. In the absence of behavioral response (a=0), the infection peak reaches approximately 6495.61 individuals at day 2.48. When adaptive behavior is incorporated (a=0.005), the peak decreases to approximately 5259.30 individuals and occurs at day 3.61. Thus, under the baseline parameter setting, adaptive behavioral feedback produces a 19.03% reduction in peak infection and delays the peak by approximately 1.13 days. These results quantitatively support the qualitative effect of behavioral adaptation predicted by the proposed model. Because the model is not calibrated to a specific outbreak, these values should be interpreted as model-based comparative outcomes rather than outbreak-specific predictions.

Peak mitigation, however, should be distinguished from cumulative transmission. Using the auxiliary cumulative-incidence variable C(t) over the same 200-day simulation horizon, we obtain C(200)=11985.63 in the absence of behavioral response and C(200)=11981.62 under adaptive behavioral response. Thus, although adaptive behavior reduces the infected peak by 19.03% and delays its occurrence by approximately 1.13 days under the baseline parameter setting, the corresponding reduction in cumulative incidence over 200 days is only approximately 0.033%. Accordingly, in this baseline scenario, the principal numerical effect of the behavioral feedback is peak mitigation and temporal redistribution of infections rather than a substantial reduction in cumulative transmission.

### Robustness Across Alternative Transmission Regimes

To assess whether the qualitative behavioral effect depends on the baseline transmission configuration, we performed an additional robustness analysis by varying the environmental half-saturation parameter *k* while keeping the remaining baseline parameters and initial conditions unchanged. We considered k=100, 1000, and 10,000, corresponding to product-form threshold values of approximately $3.33\times 10^{5}$, $3.33\times 10^{4}$, and $3.33\times 10^{3}$, respectively. These scenarios are not intended to represent calibrated low-, medium-, or high-transmission outbreaks; rather, they provide alternative illustrative transmission regimes for assessing the dependence of the behavioral effect on the baseline configuration.

For each regime, the no-behavior scenario (a=0) was compared with the baseline adaptive response (a=0.005, b=0.01). The outcomes considered were the peak number of infected individuals, time to the infected peak, cumulative incidence over 200 days, and the peak number of individuals in the isolation compartment *Q*. The latter is reported as an indicator of peak isolation burden and does not imply an independent feedback of *Q* on the transmission subsystem. The corresponding robustness results are summarized in Table 2.

Across the three illustrative transmission regimes, the direction of the behavioral effect is preserved. For the baseline behavioral intensity a=0.005, the reduction in the infected peak is approximately 19.03%, 19.10%, and 19.69% for k=100, 1000, and 10,000, respectively, while the corresponding peak delays are approximately 1.13, 1.20, and 1.55 days. Peak isolation burden is also reduced by approximately 4.24%, 4.31%, and 4.83%, respectively. In contrast, the relative reduction in 200-day cumulative incidence remains small, increasing from approximately 0.033% at k=100 to 0.092% at k=10,000.

Additional simulations with admissible behavioral intensities a=0.0025, 0.0075, and 0.01, with b=0.01 fixed, preserved the same qualitative direction of the response: stronger behavioral intensity reduced the infected peak and peak isolation burden and generally delayed the infected peak. However, the magnitude of these effects varied with the behavioral intensity and transmission configuration. Thus, the previously reported 19.03% peak reduction should be interpreted as a baseline scenario result rather than as a general quantitative property of the model.

**Figure 2:**
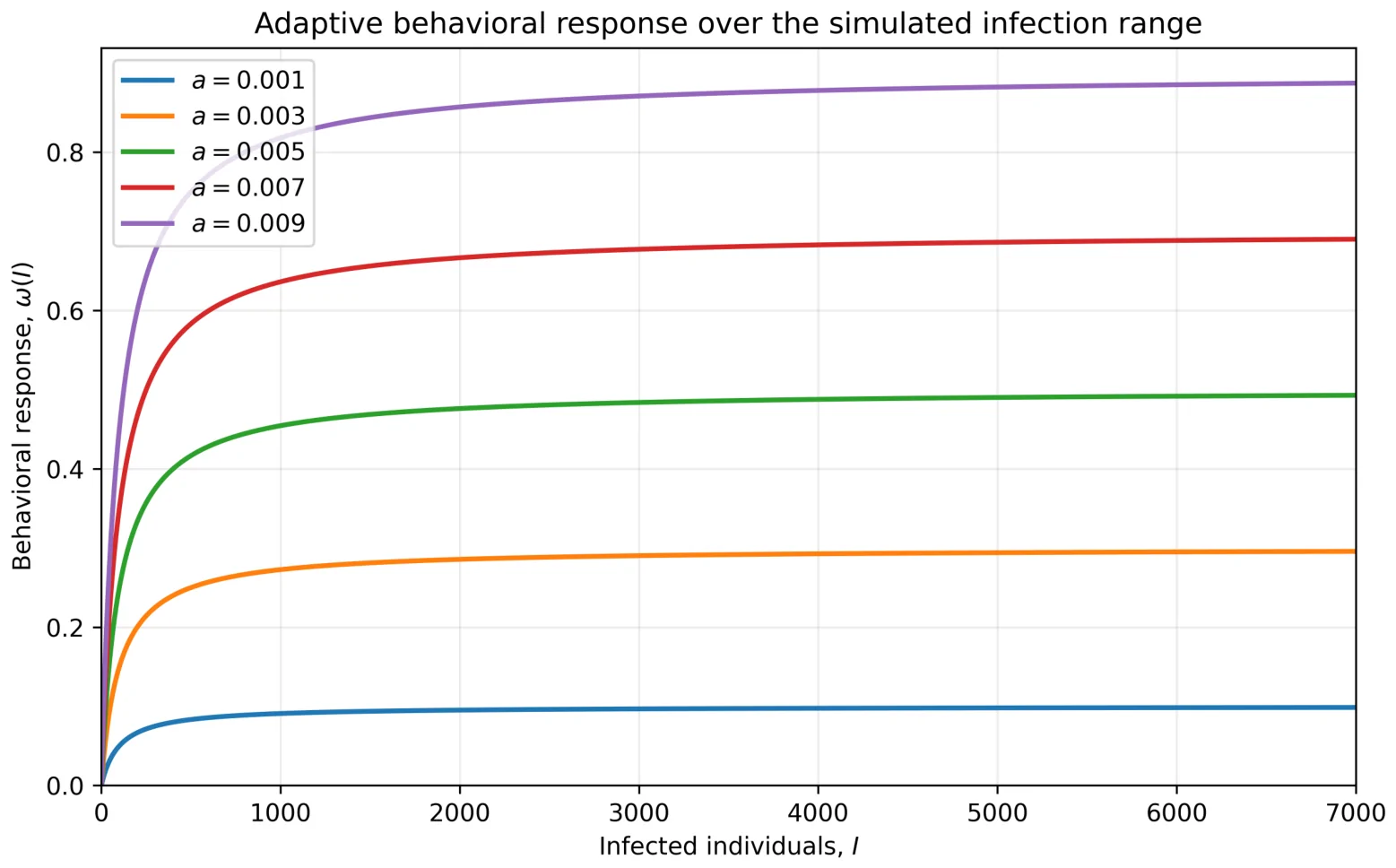
Adaptive behavioral response ω(I) for different values of the response intensity parameter *a*, with b=0.01 individual^−1^. The range 0≤I≤7000 covers the infection levels observed in the numerical simulations and illustrates the saturation of the behavioral response as the number of infected individuals increases.

Figure 2 shows the nonlinear adaptive behavioral response function used in the proposed model. As the number of infected individuals increases, the level of perceived risk and preventive behavior also increases. In this simulation, the saturation parameter *b* is kept fixed while the behavioral response intensity parameter *a* varies. The saturating shape of the curves reflects the bounded-response assumption that behavioral adaptation cannot grow indefinitely. Thus, even under high infection levels, the response approaches a maximum level determined by the parameters *a* and *b*. This figure illustrates the mathematical mechanism through which human behavior modulates the effective force of infection in the model.

## 4. Discussion of Behavioral Effects

One of the main contributions of the proposed model is the incorporation of adaptive human behavior into a classical SIQR-B framework for cholera transmission. Unlike traditional SIQR-B models, the effective transmission rate is dynamically modulated through a nonlinear risk perception function, allowing the model to capture changes in individual protective behavior as the epidemic evolves. This formulation provides a mathematically tractable framework for investigating how behavioral adaptation influences disease dynamics.

The numerical and theoretical results obtained in this study are consistent with previous investigations highlighting the important role of behavioral responses in the control of infectious diseases [13,14]. In particular, our analysis suggests that adaptive behavioral responses may reduce the epidemic peak and delay the progression of the outbreak under the assumptions of the proposed model. These findings complement previous behavioral epidemiological studies by examining adaptive risk perception within an SIQR-B framework that explicitly tracks environmental bacterial transmission and the downstream isolation burden represented by *Q*.

The behavioral response function defined in Equation (9) introduces a nonlinear feedback mechanism linking epidemic severity with individual risk perception. As the number of infectious individuals increases, the behavioral response intensifies, leading to a progressive reduction in the effective transmission rate. From an epidemiological perspective, this mechanism represents the adoption of preventive measures such as improved hygiene practices, reduced exposure to contaminated water, increased water treatment, and voluntary social distancing. The numerical simulations suggest that this adaptive behavior contributes to reducing the epidemic peak and delaying disease transmission, illustrating how behavioral changes may substantially modify the course of a cholera outbreak within the assumptions of the proposed model.

Importantly, the reduction in peak prevalence should not be interpreted as an equivalent reduction in cumulative transmission. Under the baseline simulation, adaptive behavior reduces the infected peak by 19.03% and delays its occurrence by approximately 1.13 days, whereas cumulative incidence over 200 days decreases by only approximately 0.033%. Thus, for this parameter setting, behavioral adaptation primarily redistributes infections over time and reduces short-term peak burden rather than substantially decreasing the total number of infection events over the simulation horizon. This distinction is epidemiologically relevant because peak mitigation may reduce short-term pressure on isolation and case-management capacity even when cumulative transmission changes only slightly.

Mathematically, the behavioral response function satisfies

0≤ω(I)<1,

ensuring that the effective transmission rate remains positive while decreasing as infection levels increase. The saturation structure of ω(I) represents a bounded-response assumption in which behavioral responses cannot increase indefinitely, even under high infection levels. Consequently, the proposed formulation captures the diminishing incremental effect of additional risk perception as public awareness approaches its practical limit.

The numerical simulations indicate that adaptive behavioral responses may reduce the epidemic peak and delay disease progression under the assumptions of the proposed model. These findings reinforce the importance of incorporating behavioral dynamics into mathematical models of cholera transmission. Although the present study is not intended to evaluate specific public health interventions, it suggests that population awareness and voluntary protective behaviors may contribute to mitigating disease transmission when combined with established control measures. These results suggest that behavioral adaptation and public-awareness strategies may complement established clinical, sanitation, and public-health interventions for cholera control.

The results of this study should therefore be interpreted qualitatively rather than as outbreak-specific quantitative predictions. The model has not been calibrated to epidemiological data, and its conclusions concern the mathematical and qualitative effects of adaptive behavioral feedback within the proposed framework.

## 5. Conclusions

In this work, we proposed and analyzed an extended SIQR-B mathematical model for cholera transmission incorporating adaptive human behavior through a nonlinear risk perception function. The model considers the interaction between susceptible, infected, quarantined, and recovered individuals together with the environmental concentration of *Vibrio cholerae*. The behavioral response was introduced by means of a nonlinear function that reduces the effective transmission rate as the number of infected individuals increases. Although the behavioral parameters do not modify $R_{0}$, adaptive behavior influences the transient epidemic dynamics once infection is present. Therefore, the effects of behavioral adaptation should be assessed through dynamic epidemiological outcomes rather than through the invasion threshold alone.

The qualitative analysis of the model established the positivity and boundedness of solutions, ensuring the biological feasibility of the system. Furthermore, the disease-free equilibrium and the basic reproduction number were derived using the next-generation matrix approach. Stability analysis showed that the disease-free equilibrium is locally and globally asymptotically stable whenever $R_{0}<1$.

In addition, the existence of an endemic equilibrium for $R_{0}>1$ was established, indicating the persistence of cholera transmission under appropriate epidemiological conditions. Under the baseline parameter setting, the numerical simulations showed that adaptive behavioral responses reduced the infected peak and delayed its occurrence, while producing only a small change in cumulative incidence over the 200-day simulation horizon. Therefore, these numerical results support an effect on peak burden and epidemic timing but should not be interpreted as evidence of a comparable reduction in cumulative transmission.

From the analytical perspective, the disease-free equilibrium is locally asymptotically stable when $R_{0}<1$, and the Lyapunov–LaSalle analysis extends this result to global asymptotic stability within the biologically feasible region. Thus, when the invasion threshold remains below unity, all admissible trajectories converge to the disease-free state. In contrast, when $R_{0}>1$, the model admits an endemic equilibrium, whose existence reflects the possibility of disease persistence. These analytical results provide the mathematical framework within which the effects of adaptive behavior on the transient epidemic dynamics should be interpreted.

Overall, the proposed SIQR-B framework provides a useful mathematical tool for investigating the interaction between environmental transmission and adaptive human behavior in cholera dynamics. Although the present study is theoretical in nature and is not intended to reproduce a specific outbreak, it highlights the potential influence of behavioral adaptation on epidemic evolution. Future research may include seasonal forcing, stochastic effects, spatial diffusion, vaccination strategies, parameter estimation from epidemiological data, and optimal control approaches. The modeling framework may also be extended to other environmentally transmitted infectious diseases in which behavioral adaptation plays an important role.

## Figures and Tables

**Table 1:** Model parameters, descriptions, baseline values, and units used in the numerical simulations.

Parameter	Description	Value	Unit
Λ	Recruitment rate	10	individuals/day
μ	Natural death rate	0.0001	day^−1^
$\beta_{e}$	Environmental transmission rate	0.8	day^−1^
*k*	Half-saturation constant	100	bacterial concentration
α	Recovery rate of infected individuals	0.12	day^−1^
γ	Quarantine rate	0.05	day^−1^
η	Recovery rate in quarantine	0.08	day^−1^
δ	Disease-induced death rate	0.01	day^−1^
ξ	Bacterial shedding rate	15	bacterial concentration individual^−1^ day^−1^
σ	Bacterial decay rate	0.2	day^−1^
*a*	Behavioral response intensity	0.005	individual^−1^
*b*	Behavioral saturation parameter	0.01	individual^−1^

**Table 2:** Robustness of the behavioral effect across alternative illustrative transmission regimes. The remaining parameters and initial conditions are those of the baseline simulation.

*k*	*a*	$R_{0}$	Peak *I*	Peak Time (Days)	*C* (200)	Peak *Q*
100	0	$3.33\times 10^{5}$	6495.62	2.48	11,985.63	1375.30
100	0.005	$3.33\times 10^{5}$	5259.31	3.61	11,981.62	1316.93
1000	0	$3.33\times 10^{4}$	6455.93	2.83	11,982.57	1374.81
1000	0.005	$3.33\times 10^{4}$	5223.06	4.03	11,977.90	1315.55
10,000	0	$3.33\times 10^{3}$	6211.49	4.35	11,953.80	1370.37
10,000	0.005	$3.33\times 10^{3}$	4988.75	5.90	11,942.86	1304.18

## Data Availability

No new empirical data were created or analyzed in this study. The numerical simulations were generated from the mathematical model described in the manuscript.
